# ANO1-downregulation induced by schisandrathera D: a novel therapeutic target for the treatment of prostate and oral cancers

**DOI:** 10.3389/fphar.2023.1163970

**Published:** 2023-05-18

**Authors:** SeonJu Park, Raju Das, Nguyen Xuan Nhiem, Sung Baek Jeong, Minuk Kim, Dongguk Kim, Hye In Oh, Su-Hyeon Cho, Oh-Bin Kwon, Jae-Hyeog Choi, Chul Soon Park, Song-Rae Kim, Uk Yeol Moon, Boksik Cha, Dong Kyu Choi, Sungwoo Lee, Wan Namkung, Joohan Woo, Yohan Seo

**Affiliations:** ^1^ Chuncheon Center, Korea Basic Science Institute, Chuncheon, Republic of Korea; ^2^ Department of Physiology, Dongguk University College of Medicine, Gyeongju, Republic of Korea; ^3^ Institute of Marine and Biochemistry, Vietnam Academy of Science and Technology (VAST), Hanoi, Vietnam; ^4^ Graduate University of Science and Technology, Hanoi, Vietnam; ^5^ New Drug Development Center, Daegu Gyeongbuk Medical Innovation Foundation, Daegu, Republic of Korea; ^6^ Department of Medical Device Development Center, Daegu-Gyeongbuk Medical Innovation Foundation (KMEDI hub), Daegu, Republic of Korea; ^7^ Underwood Division Economics, Underwood International College, Yonsei University, Seoul, Republic of Korea; ^8^ Department of Bio-nanomaterials, Bio Campus of Korea Polytechnics, Nonsan, Republic of Korea; ^9^ College of Pharmacy, Yonsei Institute of Pharmaceutical Science, Yonsei University, Incheon, Republic of Korea; ^10^ Channelopathy Research Center (CRC), Dongguk University College of Medicine, Goyang, Republic of Korea

**Keywords:** anoctamin 1, Schisandra sphenanthera, oral cancer, prostate cancer, protein reduction, apoptosis

## Abstract

Anoctamin 1 (ANO1), a drug target for various cancers, including prostate and oral cancers, is an intracellular calcium-activated chloride ion channel that plays various physiopathological roles, especially in the induction of cancer growth and metastasis. In this study, we tested a novel compound isolated from *Schisandra sphenanthera*, known as schisandrathera D, for its inhibitory effect on ANO1. Schisandrathera D dose-dependently suppressed the ANO1 activation-mediated decrease in fluorescence of yellow fluorescent protein; however, it did not affect the adenosine triphosphate-induced increase in the intracellular calcium concentration or forskolin-induced cystic fibrosis transmembrane conductance regulator activity. Specifically, schisandrathera D gradually decreased the levels of ANO1 protein and significantly reduced the cell viability in ANO1-expressing cells when compared to those in ANO1-knockout cells. These effects could be attributed to the fact that schisandrathera D displayed better binding capacity to ANO1 protein than the previously known ANO1 inhibitor, Ani9. Finally, schisandrathera D increased the levels of caspase-3 and cleaved poly (ADP-ribose) polymerase 1, thereby indicating that its anticancer effect is mediated through apoptosis. Thus, this study highlights that schisandrathera D, which reduces ANO1 protein levels, has apoptosis-mediated anticancer effects in prostate and oral cancers, and thus, can be further developed into an anticancer agent.

## 1 Introduction

Prostate cancer is characterized by the expression of androgen receptors and prostate-specific antigen markers that have a considerable impact on its development. Therefore, hormone therapies for prostate cancer often target these markers (McAllister et al., 2020). However, patients with prostatic small-cell neuroendocrine carcinoma frequently face restrictions in terms of their treatment, including hormone therapy (Butler and Huang, 2021), since androgen receptors and prostate-specific antigen markers are not expressed in this type of cancer. Likewise, oral cancer is the sixth most common cancer in the world (Liu et al., 2012a). The appropriate treatment for oral cancer constitutes radical surgery combined with radiation therapy and chemotherapy for progressive cancer, along with new and improved treatment methods such as 5-aminolevulinic acid photodynamic therapy. Despite these options, there is still a dire need for the discovery of new targets and therapeutic agents for the treatment of oral cancer (Karakullukcu et al., 2011). Thus, there is a need for the identification of a new target that can inhibit the development of these cancers (Tai et al., 2011; Lee et al., 2018).

Recently, it was revealed that anoctamin1 (ANO1) plays various role in cancer progression including prostate and oral cancers, and pharmacological inhibitors of ANO1 have been discovered (Seo et al., 2016; Seo et al., 2018; Galietta, 2022; Jeong et al., 2022). ANO1, which has recently emerged as a drug target, is a calcium-activated chloride channel that performs various physiological functions. The use of a pharmacological inhibitor of ANO1 in prostate PC-3 cells resulted in an anticancer effect by inducing apoptosis through the increased expression of tumor necrosis factor-α (Song et al., 2018). ANO1 inhibitors were applied on prostate cancer cells and significant antiproliferative effects were observed (Seo et al., 2017; Kim et al., 2020; Jeon et al., 2023). However, a variety of compounds act on ANO1 with only limited selectivity which poses a great challenge for the validation of ANO1 as a therapeutic target (Truong et al., 2017). Techniques for targeting specific proteins based on their structure and for understanding diverse functions of membrane proteins are flourishing astonishingly. The optimal chemical compound for targeting ANO1 can be identified using the recently discovered cryogenic electron microscopy structure of ANO1 available in the Protein Data Bank (PDB) (Paulino et al., 2017); however, many of the of properties of such compounds (for example, efficacy, selectivity, and safety) need to be tested (Liu et al., 2021).

The genus *Schisandra* (Schisandraceae), a twining shrub distributed throughout Asia and North America, comprises approximately 25–30 accepted species worldwide. Dibenzocyclooctadiene lignans and nortriterpenoids are the main chemical components of this genus (Li et al., 2003; Luo et al., 2012; Li et al., 2013; Shi et al., 2015). Previous phytochemical studies on *Schisandra sphenanthera* have resulted in the identification of diverse chemical structures, including highly oxygenated nortriterpenoids, diaryldimethylbutane, dibenzocyclooctadiene lignans, and neolignans (Peng et al., 2019; Mai et al., 2021). Interestingly, the *Schisandra* lignans mentioned above demonstrate a wide range of pharmacological activities, such as cytotoxic, hepatoprotective, neuroprotective, and cognitive-enhancement (Wang et al., 2008; Li et al., 2013; Jiang et al., 2015; Sowndhararajan et al., 2018; Mai et al., 2021).

In the present study, we investigated the possible medicinal effects of phytochemicals obtained from *S. sphenanthera* occuring owing to the inhibition of ANO1, and the physiological effects of schisandrathera D on prostate and oral cancer cells. Through these investigations, we aimed to demonstrate the anticancer effects of this compound.

## 2 Materials and methods

### 2.1 Plant material

The leaves of *S. sphenanthera* Rehder & E. H. Wilson were collected from the Kon Tum province in February 2017 and identified by Dr. Nguyen, The Cuong, Institute of Ecology and Biological Resources. A voucher specimen (NCCT-P78) was deposited at the Institute of Marine Biochemistry in Vietnam.

### 2.2 Extraction and isolation

The dried powdered *S. sphenanthera* (5 kg) was extracted thrice with methanol (MeOH; 10 L each time), in an ultrasonic bath, at room temperature. Removal of the solvent under vacuum evaporation resulted in 220 g of crude MeOH extract (4.4% yield), which was suspended in water and partitioned with dichloromethane and then ethyl acetate, resulting in 22.3 g dichloromethane (0.4% yield), 70.0 g ethyl acetate (1.4% yield), and 89.0 g of a water layer (1.8% yield).

The ethyl acetate crude fraction was chromatographed on a silica gel column using a stepwise gradient of hexane/ethyl acetate, to yield seven fractions, E1–E7. E1 was first purified using silica gel CC, and then eluted with hexane/ethyl acetate (5/1, v/v), to yield six fractions, E1.1–E1.6. E1.2 was further purified using preparative high-performance liquid chromatography (HPLC) and as an eluent, MeOH/water (4/1, v/v) to yield schisandrathera D (3.5 mg) (Mai et al., 2021).

### 2.2 Cell culture

Fisher rat thyroid (FRT) cells stably expressing ANO1 and cystic fibrosis transmembrane conductance regulator (CFTR) were kindly provided by Dr. Alan Verkman (University of California, San Francisco, CA, United States) and cultured in Coon’s modified F12 medium. PC-3 (human prostate cancer cell line) and CAL-27 (human oral adenosquamous carcinoma cell line) cells were cultured in Roswell Park Memorial Institute-1640 (RPMI 1640) or Dulbecco’s modified Eagle’s medium. All the media contained 10% fetal bovine serum, 2 mM L-glutamine, 100 U·mL^−1^ penicillin, and 100 μg mL^−1^ streptomycin.

### 2.4 Construction of ANO1-knockout (KO) cells

The PLentiCRISPRv2 vector containing Cas9 and CRISPR guide RNA targeting ANO1 (CCT​GAT​GCC​GAG​TGC​AAG​TA) (clone ID: X35909) was purchased from Genscript (Piscataway, NJ, United States). In total, 1,500 ng of CRISPR plasmid, 1,200 ng of packaging plasmid (psPAX2), and 400 ng of envelope plasmid (pMD2.G) were co-transfected into HEK293T cells cultured in 6-well plates. The supernatant containing lentiviral particles was collected 48 h post-transfection and filtered using a 0.45 μm syringe filter. The cells were treated overnight with lentiviral particles mixed with fresh medium at a ratio of 1:1, in 24-well plates. The ANO1-KO cells were then selected using puromycin (Sigma-Aldrich, St. Louis, MO, United States), 72 h after virus transduction.

### 2.5 Yellow fluorescent protein (YFP) fluorescence quenching analysis

FRT cells stably expressing both the YFP variant (YFP-H148Q/I152L/F46 L) and ANO1 were plated into 96-well plates, at a density of 2 × 10^3^ cells per well. After 48 h of incubation, the cells in each well were washed twice with phosphate-buffered saline (PBS), following which they were incubated for 10 min with the test compounds dissolved in PBS. The YFP fluorescence in each well was measured every 0.4 s for 5 s, using the FLUO star^®^ Omega microplate reader (BMG Labtech). ANO1 acts as an iodide as well as a chloride channel. ANO1 activation causes iodide influx. To measure ANO1-mediated iodide influx, 100 μL of 70 mM iodide solution with 100 μM ATP was injected into each well, 1 s after initiation of measurement. The inhibitory effect of the test compounds on ANO1 activity was measured in terms of the initial iodide influx rate, which was determined from the initial slope of the decrease in fluorescence after ATP injection.

### 2.6 Measurement of short-circuit currents

Snapwell™ inserts containing ANO1- and CFTR-expressing FRT cells were mounted onto Ussing chambers (Physiologic Instruments, San Diego, CA, United States). The basolateral bath was filled with a HCO_3_^−^-buffered solution containing 120 mM NaCl, 5 mM KCl, 1 mM MgCl_2_, 1 mM CaCl_2_, 10 mM D-glucose, 2.5 mM HEPES, and 25 mM NaHCO_3_ (pH 7.4), while the apical bath was filled with a half-Cl^−^ solution. In the half-Cl^−^ solution, 65 mM NaCl in the HCO3^−^-buffered solution was replaced with sodium gluconate. The basolateral membrane was permeabilized with 250 μg mL^–1^ amphotericin B. The cells were bathed for 20 min and aerated with 95% O_2_/5% CO_2_, at 37°C. ATP was applied to the apical membrane to activate ANO1, while forskolin was applied to the apical membrane to activate CFTR. Then, test compound was applied to both apical and basolateral bath solutions, 20 min before ANO1 and CFTR activation. Apical membrane currents were measured using an EVC4000Multi-Channel V/I Clamp (World Precision Instruments, Sarasota, FL, United States) and PowerLab 4/35 (AD Instruments, Castle Hill, Australia). Data were analyzed using LabChart Pro 7 (AD Instruments). The sampling rate was 4 Hz.

### 2.7 Measurement of intracellular calcium levels

FRT cells were cultured in 96-well black-walled microplates and loaded with Fluo-4 NW, according to the manufacturer’s protocol (Invitrogen, Carlsbad, CA, United States). Briefly, the cells were incubated with 100 μL assay buffer (1 × Hanks’ balanced salt solution with 2.5 mM probenecid and 20 mM HEPES) containing Fluo-4 NW. After 1 h of incubation, the 96-well plates were transferred to a plate reader for the fluorescence assay. Fluo-4 fluorescence was measured using a FLUOstar^®^ Omega microplate reader equipped with syringe pumps and custom Fluo-4 excitation/emission filters (485/538 nm).

### 2.8 Western blot analysis

Protein samples (80 μg) were separated using 4%–12% Tris-Glycine-PAG Pre-Cast Gel (Koma Biotech, South Korea) and transferred to polyvinylidene fluoride membranes. Blocking was performed using 5% bovine serum albumin (BSA) in tris-buffered saline with 0.1% Tween 20 for 1 h. The membranes were then incubated with the corresponding primary antibodies, including anti-ANO1 (Abcam, United Kingdom) and anti-β-actin (Santa Cruz Biotechnology, United States), followed by incubation with horseradish peroxidase-conjugated anti-secondary IgG antibodies (Enzo Life Science) for 1 h. Finally, visualization was performed using the ECL Plus Western Blotting System (GE Healthcare).

### 2.9 Cell viability assay

MTS cell proliferation assay was performed using CellTiter 96^®^ AQueous One Solution Cell Proliferation Assay Kit (Promega). PC-3 and CAL-27 cells were cultured in 96-well plates containing a medium supplemented with 3% fetal bovine serum for 24 h. Once the cells reached approximately 20% confluence, the compounds (0.03–300 μM) or vehicle (DMSO) were added to the medium, which was replaced with a fresh medium every 24 h. After 48 h of treatment, the medium was completely removed and MTS assay was performed, as recommended in the supplier’s protocol. The absorbance of formazan was measured at a wavelength of 490 nm, using an Infinite M200 microplate reader (Tecan).

### 2.10 Molecular docking analysis

Before docking, ligand preparation was carried out using Schrödinger Suite 2017–1. Following that, the 3D structure of the selected ligand was drawn using the ChemDraw software, while compound Ani9 was downloaded from the PubChem database (Jeong et al., 2022). For precise three-dimensional ligand models and conformational sampling, all compounds were exposed to the LigPrep module in Schrödinger Suite while maintaining a pH of 7.0 ± 2.0 for structure refinement, proper chiral formation, and ionization prediction. The entire system was run under the OPLS3 force field, with the implementation of the Epik algorithm script (version 4.4) (Shelley et al., 2007). For each ligand, a total of 32 possible stereoisomers were set. Here, the protein preparation wizard of Schrodinger suite was used to prepare the structure before docking. Due to the absence of a human crystallographic structure, we selected two cryogenic structures (PDB: 5OYB, 6BGJ), which correspond to *Mus Musculus*. Both structures were passed through the optimization and minimization steps. Additionally, proper hydrogen additions, bond order correction, and deletion of water molecules were confirmed during the preparation. After that, grid generation of the protein-ligand-binding site was also defined by following the glide-grid generation protocol in the Schrödinger Suite. For the preliminary quest of receptor-ligand-binding insights and selection of an accurate binding pose, extra precision (XP) docking was carried out to obtain a more reliable score than that obtained using standard precision (SP) docking, wherein ligands were allowed to move flexibly into the binding core (Halgren et al., 2004). As indicated by the methodology, the candidate proteins were docked with two selected ligands following the constraint minimization process. Before docking, Van der Waals scaling factor and charge cutoff were kept constant at 0.80 and 0.15, respectively, to soften the potential for nonpolar parts of the ligand. After the final docking, the lowest scores based on the best orientations were sorted for further study.

### 2.11 Prime molecular mechanics—generalized born surface area (MM-GBSA)

To evaluate the actual binding free energy of the complexes generated upon the docking simulation, the complexes were subjected to MM-GBSA analysis, a mixed methodology of the prime MM-GBSA protocol. MM-GBSA is a combined protocol wherein the OPLS3 force field is used to predict the molecular mechanics energy and the surface-generalized Born solvation model is used for polar solvation calculations. A non-polar solvation term composed of a non-polar solvent-accessible surface area and van der Waals interactions also constitutes a part of the total MM-GBSA calculation process (Vijayakumar et al., 2011). After the post-energy calculation, the best hits were identified as those with higher negative energy values. The total free energy of binding was calculated using the following equation: ΔGbind = Gcomplex − (Gprotein + Gligand), where G = molecular mechanics energy + GSGB + non-polar solvation term.

### 2.12 Caspase-3 activity assay

PC-3 and CAL-27 cells were cultured in 96-well plates until they reached 30% confluence. Next, test compounds (0, 10, and 30 μM), Ac-DEVD-CHO (10 μM), a caspase-3 inhibitor, and Ani9 (10 μM) were added to the corresponding wells. After 24 h, each well was washed with PBS and incubated at room temperature with 100 μL PBS containing 1 μM NucView^®^ 488 caspase-3 substrate. After 30 min of incubation, the cells were stained with 1 μM Hoechst 33342. The fluorescence of NucView^®^ 488 and Hoechst 33342 was measured using a FLUOstar^®^ Omega microplate reader and multicolored images were documented using a Lionheart FX Automated Microscope (BioTek, Winooski, VT, United States).

### 2.13 Human cleaved PARP-1 activity assay

Human cleaved PARP-1 activity assay was performed according to the manufacturer’s instructions (#ab174441, Abcam, United Kingdom). PC-3 and CAL-27 cells were cultured in 6-well plates until they reached 80% confluence. Test compounds were added for 24 h and 5 × 10^7^ cells were lysed using the cell extraction buffer for 20 min. Supernatants were collected after the cells were centrifuged (13,000 rpm) for 20 min at 4°C. Next, 100 μg protein/50 μL buffer was added to each well with an antibody cocktail containing a capture antibody and detector antibody and incubated for 1 h. The wells were washed twice with 1 x wash buffer and then TMB development solution was added for 10 min. Finally, the stop solution was added, and optical density (O.D.) was measured at 450 nm using a microplate reader (Synergy™ Neo, BioTek).

### 2.14 Cell-cycle analysis

PC-3 cells were seeded 2 × 10^5^ cells/well in 100-phi culture plates and treated with the test compound for 24 h. Cells were fixed with cold 70% ethanol and incubated for 2 h at −20°C. Ethanol was removed and the cells were re-suspended in propidium iodide/Triton X-100/DNase-free RNase A staining solution for 30 min. The cell-cycle distribution was analyzed using Gallios (Beckman Coulter). A total of 2 × 10^3^ cells per group were analyzed.

### 2.15 Statistical analysis

All experiments were conducted independently at least thrice. The results for the multiple trials have been presented as mean ± standard error. Statistical analysis was performed using Student’s t-test or analysis of variance, as appropriate. Statistical significance was set to *p* < 0.05. Prism software (GraphPad) was used to plot the dose-response curve and calculate the IC_50_ values.

## 3 Results

### 3.1 Identification of compounds from *S. sphenanthera*

**FIGURE 1:**
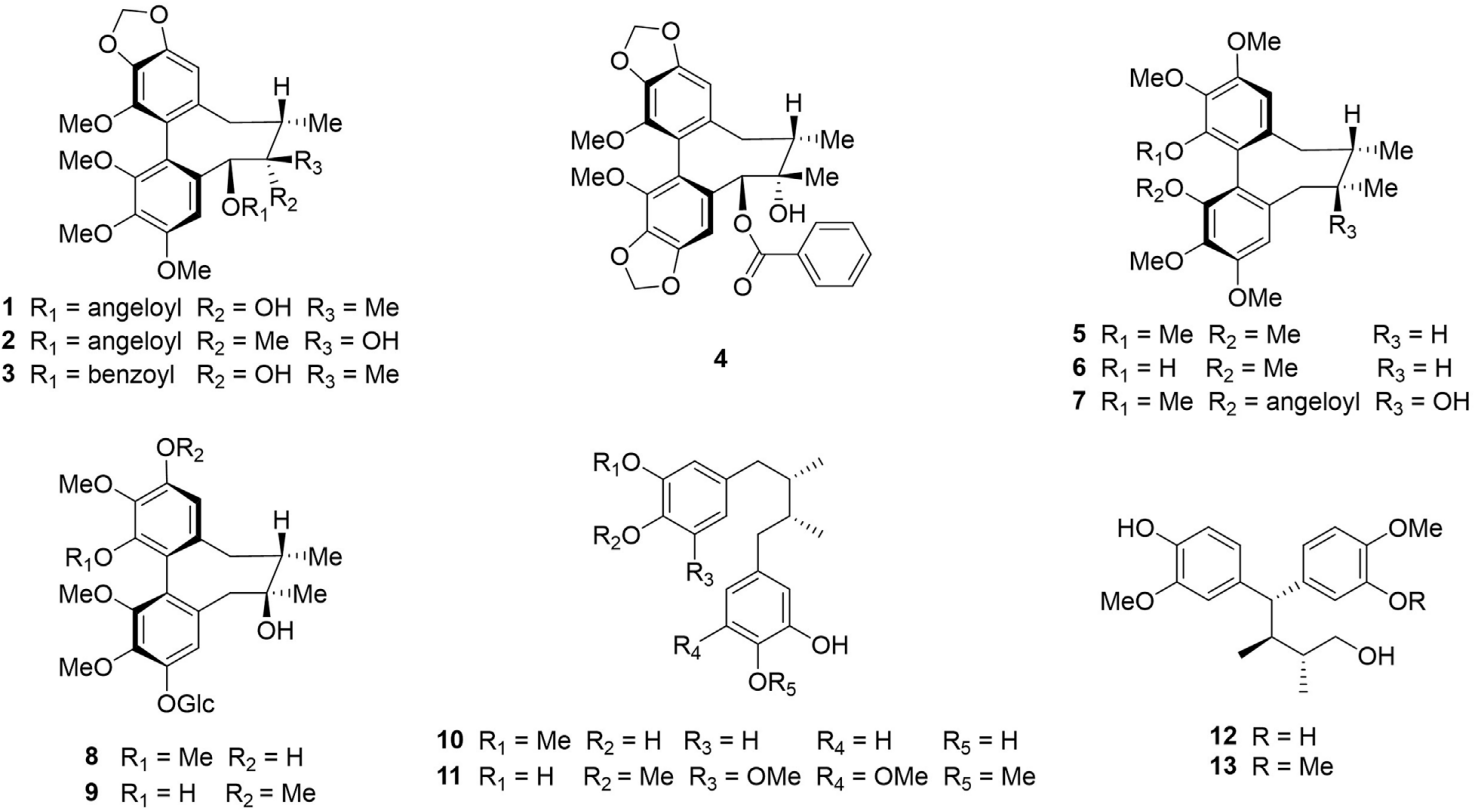
Chemical structures of compounds 1–13: schisantherin B (**1**), tigloylgomisin P (**2**), schirubrisin B (**3**), schisantherin D **(4)**, (+)-deoxyschizandrin **(5)**, (+)-gomisin K3 **(6)**, angeloylgomisin H **(7)**, 3, 7-dihydroxy-1, 2, 13, 14-tetramethoxydibenzocyclooctadiene 12-O-*β*-D-glucopyranoside (**8**), schisandrathera B (**9**), meso-dihydroguaiaretic acid (**10**), schisandrathera D (**11**), 4, 4-bis (4-hydroxy-3-methoxyphenyl)-2, 3-dimethyl-1-butanol (**12**), and schisandrathera C (**13**).

In previous studies, we isolated thirteen dibenzocyclooctadiene lignans and neolignans, namely schisantherin B (McAllister et al., 2020), tigloylgomisin P (Butler and Huang, 2021), schirubrisin B (Lee et al., 2018), schisantherin D (Tai et al., 2011), (+)-deoxyschizandrin (Liu et al., 2012b), (+)-gomisin K3 (Karakullukcu et al., 2011), angeloylgomisin H (Sung et al., 2021), 3, 7-dihydroxy-1, 2, 13, 14-tetramethoxydibenzocyclooctadiene 12-O-β-D-glucopyranoside (Vitório et al., 2020), schisandrathera B (Jeong et al., 2022), meso-dihydroguaiaretic acid (Galietta, 2022), schisandrathera D (Seo et al., 2018), 4,4-bis(4-hydroxy-3-methoxyphenyl)-2,3-dimethyl-1-butanol (Seo et al., 2016), and schisandrathera C (Song et al., 2018), from the leaves of *S. sphenanthera* (Figure 1). Their chemical structures, including absolute configurations were determined extensively by HR-ESI-MS, NMR, and ECD spectra and comparing their NMR data with that in reported literature (Mai et al., 2021).

### 3.2 Cell-based high-throughput screening (HTS) for the identification of a novel natural compound that inhibits ANO1 channel

**FIGURE 2:**
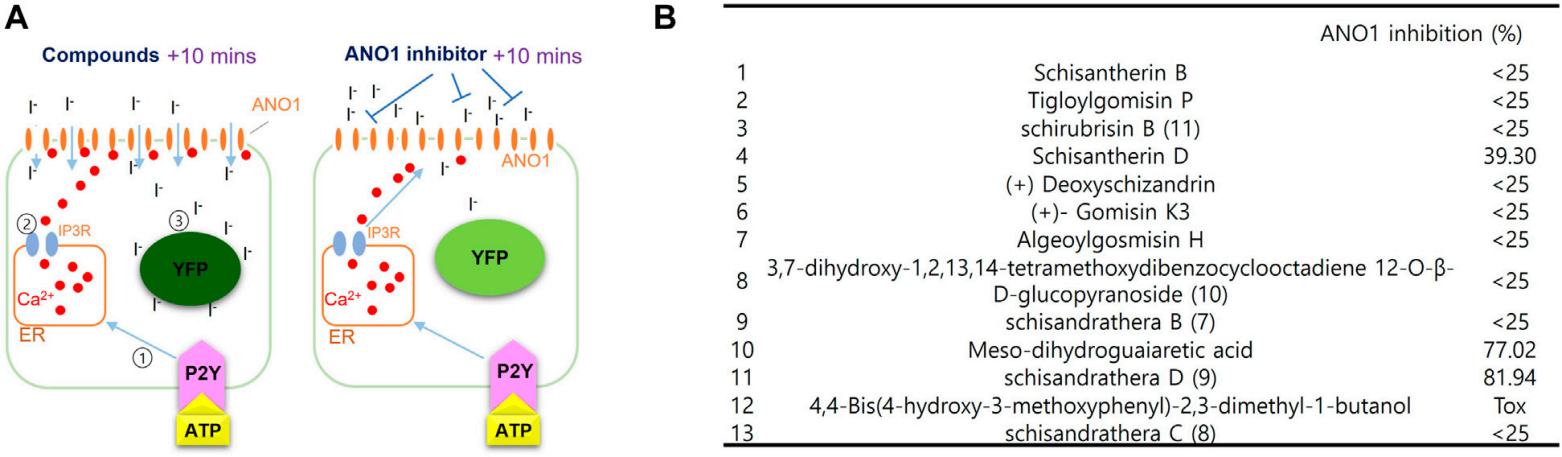
Identification of schisandrathera D using yellow fluorescent protein (YFP)-based high-throughput screening **(A)** A schematic representation of the cell-based YFP-reduction assay. Activation of ATP-induced P2Y receptor increases calcium levels, thereby causing activation of ANO1 channel, resulting in an influx of iodide, which quenches YFP. **(B)** Effects of the compounds given in the table (each used at a concentration of 10 μM) on ANO1 were assessed in terms of YFP fluorescence. The results have been presented as mean ± standard deviation (*n* = 5).

The effect of substances extracted from schisantherin on inhibiting ANO1 was measured using a cell-based screening system. As shown in Figure 2, upon treatment with adenosine triphosphate (ATP), there is an increase in the levels of intracellular calcium, which leads to the flow of iodine into the cell through ANO1 channel activation because ANO1 channel also acts as an iodide channel. The intracellular iodine binds to the mutant YFP and strongly reduces its fluorescence. However, if an ANO1 inhibitor inhibits the ANO1 channel and blocks the influx of iodine, there is no decrease in the YFP fluorescence (Figure 2A). Upon screening of the substances isolated from schisantherin for their ANO1-inhibitory effect, schisandrathera D was found to have the strongest effect (Figure 2B).

### 3.3 Schisandrathera D is a selective ANO1 inhibitor

**FIGURE 3:**
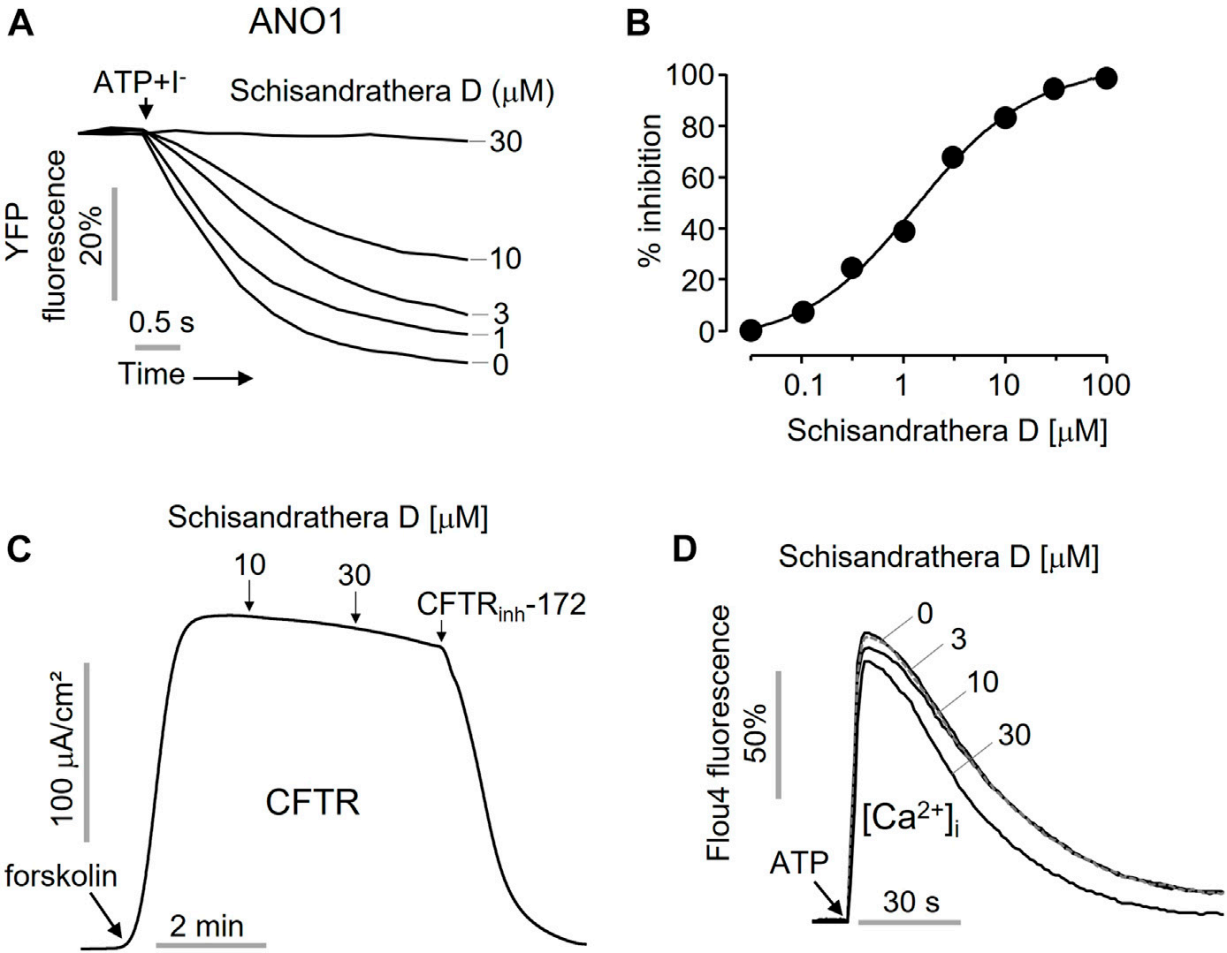
Characterization of the novel ANO1 inhibitor, schisandrathera D **(A)** Effects of the indicated concentrations of schisandrathera D on yellow fluorescent protein (YFP) fluorescence, upon 10-min treatment **(B)** Schisandrathera D inhibited Anoctamin 1 in a dose-dependent manner. **(C)** Effect of schisandrathera D on forskolin-induced cystic fibrosis transmembrane conductance regulator channel activity. **(D)** The intracellular calcium levels in Fisher rat thyroid (FRT) cells treated with schisandrathera D, as measured using Fluo-4 NW assay kit. The results have been represented as a representative trace (mean ± standard error, *n* = 5).

As shown in Figure 3, to check whether the inhibition of the ANO1 channel function by schisandrathera D is selective, FRT cells expressing ANO1 and YFP at different concentrations were treated with schisandrathera D for 20 min. Then, the treatment with ATP and iodide induced a decrease in the measured YFP fluorescence. Schisandrathera D inhibited the function of ANO1 in a concentration-dependent manner, with an IC_50_ of 5.24 µM (Figures 3A, B). In addition, to determine whether schisandrathera D inhibits the CFTR chloride channel, CFTR-expressing FRT cells were differentiated for 6 days, and the short-circuit currents were measured using an Ussing chamber. When cells are treated with forskolin, an adenylyl cyclase activator, there is an increase in intracellular cyclic adenosine monophosphate levels, which in turn causes protein kinase A to activate CFTR. As shown in Figure 3C, schisandrathera D did not inhibit forskolin-induced CFTR activity, whereas CFTR-_inh_172, a selective CFTR inhibitor, inhibited CFTR-induced current. Since ANO1 is also activated by low calcium levels (Arreola et al., 2022), we confirmed whether schisandrathera D had an effect on the ATP-mediated increase in intracellular calcium signaling. Since schisandrathera D had no effect on intracellular calcium levels at concentrations of up to 30 μM, it can be argued that schisandrathera D selectively inhibits ANO1.

### 3.4 Prediction of binding sites on the ANO1 protein

ANO1 channels are opened by voltage-dependent or voltage-gated calcium channels, and the secretion of chloride ions by ANO1 is highly dependent on calcium levels (Segura-Covarrubias et al., 2020). There are several calcium-binding sites, and when calcium is attached to its transmembrane site, the channel becomes depolarized, thereby leading to opening of pores (Ji et al., 2021). In the present study, we performed molecular docking to determine whether schisandrathera D acts at a location similar to the binding site of calcium. Glide XP docking was performed to check the molecular interaction and binding affinity, and to investigate the structure of the protein-ligand complexes after docking. Two cryogenic electron microscopy structures (PDB IDs: 5OYB and 6BGJ) were used to identify intermolecular interactions with the selected ligand after molecular docking. To date, the exact ligand-binding mechanism of ANO1 is unknown. Therefore, according to a previous study based on mutagenesis studies, putative residues involved in calcium-binding were considered as ligand-binding sites in this study (Vijayakumar et al., 2011).

**FIGURE 4:**
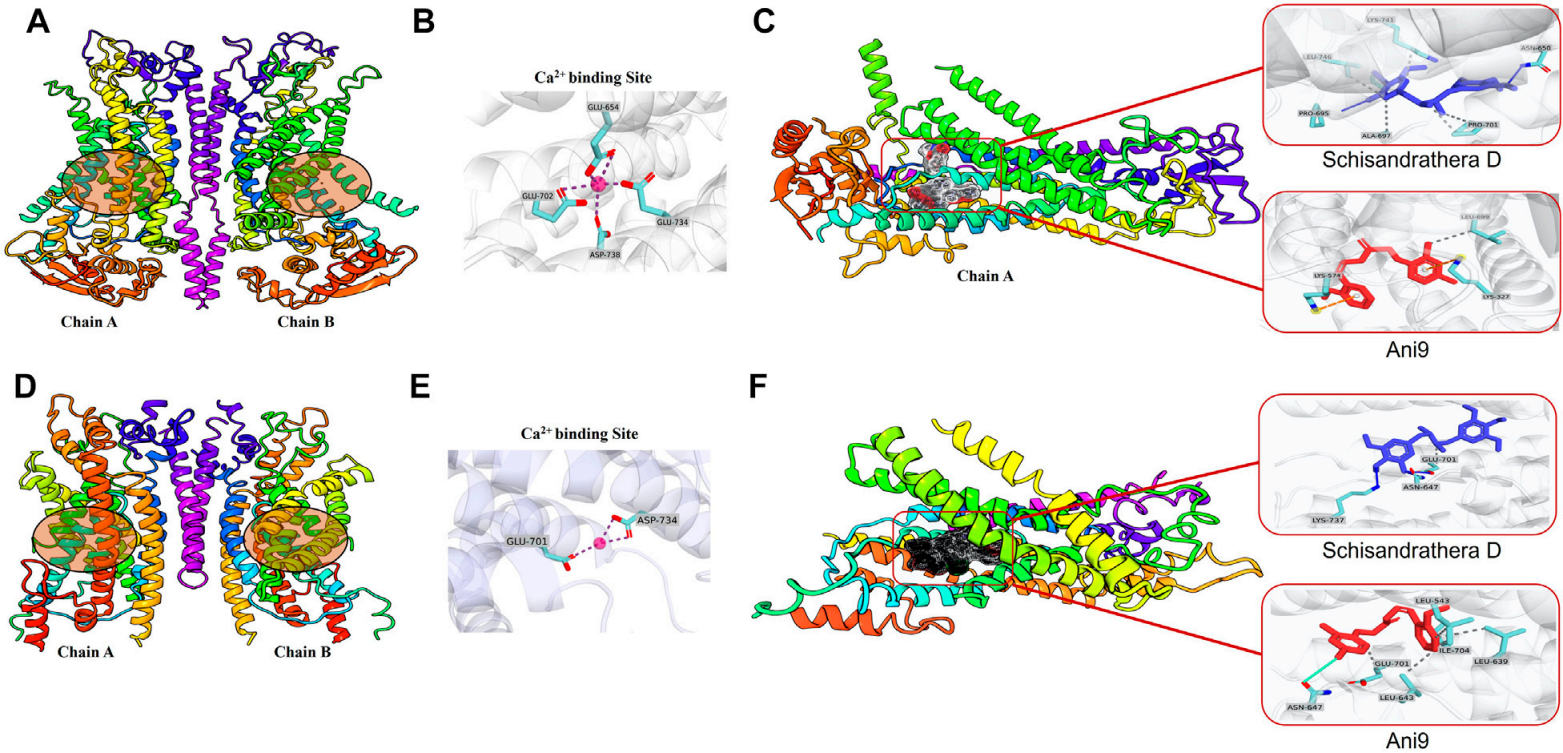
Intermolecular interaction analysis of schisandrathera D and Ani9 with two different Anoctamin 1 (ANO1) structures **(A)** A dimer of mANO1 channel containing chains A & B (PDB: 5OYB). The circular area denotes the Ca^2+^-binding site. **(B)** The Ca^2+^-binding site formed by residues Glu645, Glu702, Glu734, and Asp738. **(C)** Representative images of schisandrathera D and Ani9 (control) bound to the active site of mANO1 (PDB: 5OYB). **(D)** A dimer of mANO1 channel containing chains A & B (PDB: 6BGJ). The circular area denotes the Ca^2+^-binding site. **(E)** The Ca^2+^-binding site formed by residues Glu701 and Asp734. **(F)** Representative images of schisandrathera D and Ani9 (control) bound to the active site of mANO1 (PDB: 6BGJ).

Binding of the two ligands to the ANO1 binding core is shown in Figure 4 and the supplementary file (Supplementary Table S1). The docking results showed that schisandrathera D generated numerous hydrogen and hydrophobic contacts (Figures 4A–C). Schisandrathera D established hydrogen bonds with His650, His661, and His695 and interacted hydrophobically with Ala697, Pro701, Lys741, and Leu746. Ani9, however, formed a hydrophobic interaction with residue Leu699 and a π stacking interaction with Lys327 and Lys574. In contrast, schisandrathera D showed fewer hydrogen and hydrophobic interactions with the other structure (Figures 4D–F). As shown in Figure 4F, schisandrathera D formed hydrogen bonds with Asn647, Glu701, and Lys737 in this case, and a hydrophobic contact with Glu701. Ani9 formed five hydrophobic interactions with the residues Lue543, Lue639, Lue643, Glu701, and Ile704, and a halogen contact with the residue Asn647. Based on the above interaction, the molecular docking and MM-GBSA scores of schisandrathera D with the two distinct structures suggested better binding mechanisms than those of the control, Ani9. The molecular docking and MM-GBSA scores are provided in the supplementary file (Supplementary Table S2).

### 3.5 Schisandrathera D reduces ANO1 protein levels and cell viability

**FIGURE 5:**
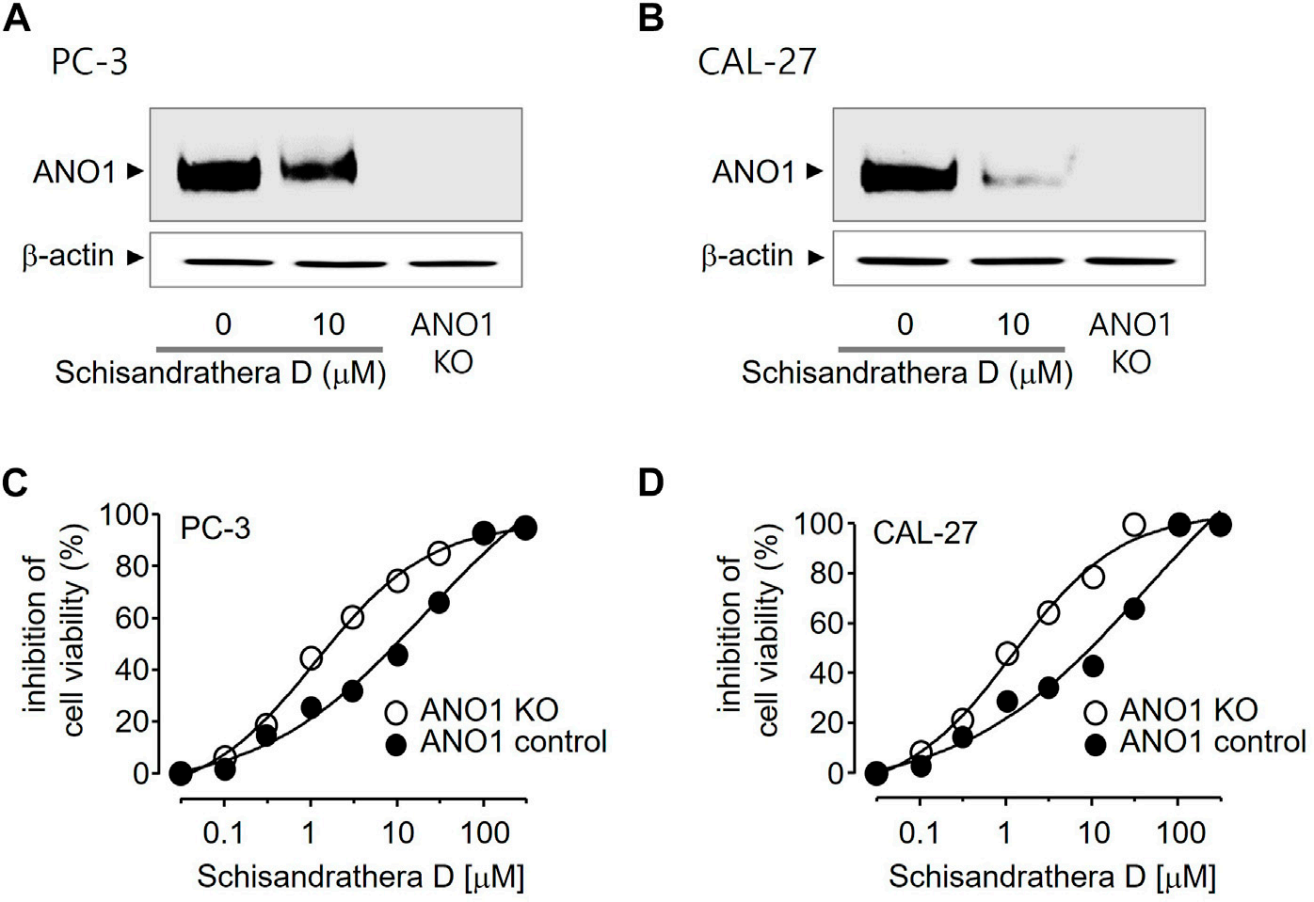
Effects of schisandrathera D on Anoctamin 1 (ANO1) protein expression and cell viability **(A,B**) PC-3 and CAL-27 cells were cultured with 10 μM schisandrathera D for 24 h. ANO1 expression in PC-3/CAL-27 ANO1-KO cells. The loading control was *ß*-actin. **(C,D)** Effects of the indicated concentrations of schisandrathera D on cell viability of PC-3 or PC-3 ANO1-KO cells and CAL-27 or CAL-27 ANO1-KO cells. The data have been represented as mean ± standard deviation (*n* = 5).

Pharmacological inhibition of ANO1 inhibits the growth of various cancer cell types. To determine whether schisandrathera D inhibits the growth of prostate and oral cancers through a decrease in ANO1 protein levels, PC-3 and CAL-27 cells were treated with schisandrathera D and the change in the level of ANO1 protein was measured. Schisandrathera D strongly decreased the ANO1 protein levels in both cell lines (Figures 5A, B). In addition, we also generated ANO1-KO cells using CRISPR-Cas9 system. These genetically engineered cells were also treated with different concentrations of schisandrathera D, following which cell viability was measured. Schisandrathera D decreased cell viability in a concentration-dependent manner in cells with high ANO1 levels, but there was no such inhibitory effect in ANO1-KO cells (Figures 5C, D).

### 3.6 Assessment of the apoptotic effect of schisandrathera D

**FIGURE 6:**
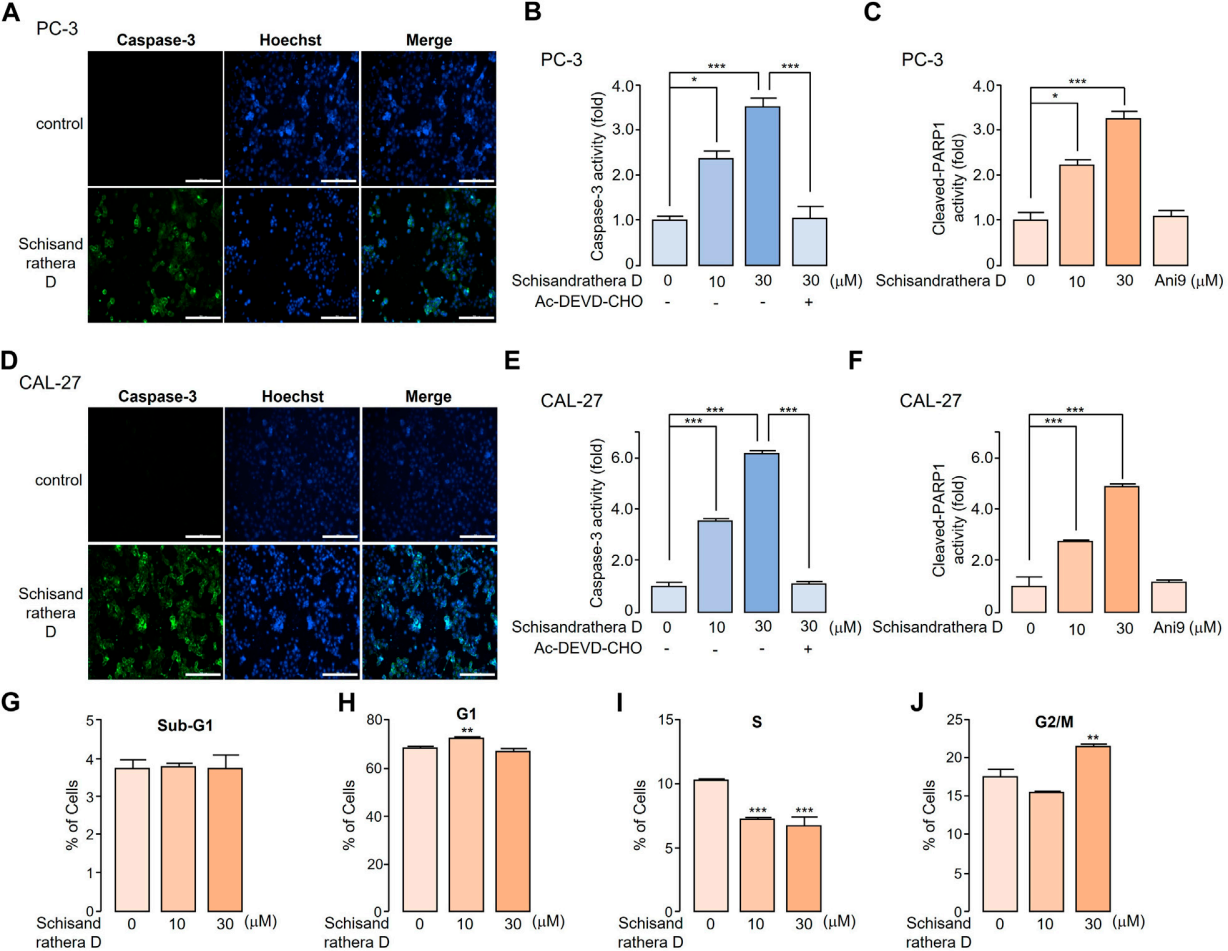
Apoptotic effect of schisandrathera D **(A)** PC-3 and **(D)** CAL-27 cells were cultured with 10 and 30 μM schisandrathera D for 6 h, following which they were treated with 1 μM caspase-3 substrate and 1 μM Hoechst 33342 for 30 min. The apoptotic cells were stained with green fluorescence, while nuclei were stained with Hoechst 33342 (blue). **(B)** PC-3 and **(E)** CAL-27 cells were cultured with the indicated concentrations of schisandrathera D for 6 h, and then treated with 1 μM caspase-3 substrate for 30 min. Caspase-3 was inhibited by means of treatment with 10 μM Ac-DEVD-CHO (mean ± standard deviation (S.D.), *n* = 5). Levels of cleaved poly-ADP ribose polymerase 1 in **(C)** PC-3 and **(F)** CAL-27 cells incubated with the indicated concentrations of schisandrathera D and Ani9 for 6 h (mean ± S.D., *n* = 5). **(G–J)** PC-3 cells were exposed to 10 and 30 µM of schisandrathera D for 24 h. Distribution of cell population was analyzed using propidium iodide staining followed by flow cytometry analysis. Data have been represented as mean ± S.D. of three different experiments. *p < 0.05, **p < 0.01, and ***p < 0.001, assessed using Student’s unpaired *t*-test.

Reduction or pharmacological inhibition of ANO1 induces apoptosis. We treated PC-3 and CAL-27 cells expressing high levels of ANO1 with schisandrathera D and determined if this treatment induced apoptosis. Schisandrathera D significantly increased the activity of caspase-3 and poly (ADP-ribose) polymerase 1 cleavage, which serve as two hallmarks of apoptosis, while Ani9 did not increase the latter (Figure 6). Although Ani9 is a known ANO1 inhibitor, its effect on reducing the ANO1 protein levels was weak. Schisandrathera D, however, induced an apoptotic effect and G2M arrest by strongly decreasing the ANO1 protein levels.

## 4 Discussion

Although the physiological functions of ANO1 are diverse and include secretion of chloride ions and the development of cancer (Hwang et al., 2009; Cho et al., 2012; Huang et al., 2012; Ji et al., 2019), the inhibition of the function of ANO1 channel and reduction of its protein expression has therapeutic effects in head and neck cancers, and in prostate cancers (Ayoub et al., 2010; Liu et al., 2012a; Duvvuri et al., 2012). In recent studies, ANO1 has been proposed as a potential therapeutic target for several cancers, including OSCC. No molecules which can ameliorate OSCC via ANO1 inhibition are known to date (Ruiz et al., 2012; Britschgi et al., 2013; Li et al., 2014; Seo et al., 2015). Therefore, it is necessary to find novel molecules that can reduce ANO1 channel function or ANO1 protein levels. In this study, we found that schisandrathera D reduced the ANO1 channel function in a dose-dependent manner. Interestingly, schisandrathera D neither inhibited CFTR activity, nor the ATP-induced changes in calcium levels, thereby behaving as a selective ANO1 inhibitor (Figures 2, 3).

To date, several ANO1 inhibitors have been developed (Galietta et al., 2001; Namkung et al., 2010; Seo et al., 2016; Seo et al., 2018). Therefore, several research groups tried to develop specific ANO1 inhibitors, starting from hit to lead chemical compounds via lead optimization (Seo et al., 2018; Wang et al., 2022). However, they have not reached clinical trials because they generally have low *in vitro* stability and *in vivo* efficacy (Choi et al., 2021; Wang et al., 2021; Zhang et al., 2021).

Therefore, natural products may provide a breakthrough in this field of research, being effective ANO1 inhibitors; furthermore, such products may be safer. Thus, many researchers are actively conducting studies to develop anticancer drugs based on natural products (Kim and Kim, 2018; Spradlin et al., 2019).

We have demonstrated for the first time that the natural compound schisandrathera D, isolated from the leaves of *S. sphenanthera*, strongly inhibits the function of ANO1 channels by binding to the ANO1 protein site (Figure 4). Schisandrathera D appears to bind to a specific region of the ANO1 protein with high absolute binding free energy and prevent its activation by calcium (Tai et al., 2011). Furthermore, there appears to be a high probability that this binding to ANO1 is beneficial for reducing its protein levels. Ani9 binds to ANO1 protein with low binding free energy, but does not reduce the ANO1 protein levels (Seo et al., 2021). However, since ANO1 activation can be caused by several molecular factors such as internal levels of calcium, calcium binding protein (CaM) and inositol trisphosphate (Hawn et al., 2021), the mechanism underlying the schisandrathera D-mediated reduction in protein levels requires further study.

Schisandrathera D seems to be an effective anticancer therapeutic option, acting through ANO1 inhibition. Treatment of PC-3 and CAL-27 cells with dose-dependent concentrations of schisandrathera D reduced their viability; however, this ANO1-dependent reduction in cell viability was more marked in PC-3 and CAL-27 cells that expressed high levels of ANO1. However, schisandrathera D did inhibit cell viability weakly in ANO1 KO-PC-3 and -CAL-27 cells. This suggests that the anticancer mechanism of schisandrathera D is, at least partly, mediated by the reduction of ANO1 expression. Moreover, Ani9 (the only functional ANO1 inhibitor) did not induce apoptosis. Similarly, luteolin, an ANO1 inhibitor, displays anticancer effects through the downregulation of ANO1 in cancer cells (Seo et al., 2017). Therefore, it is possible that the downregulation of ANO1 by schisandrathera D represents an important anticancer mechanism. However, future studies should focus on the comparative assessment of the absorption and metabolic rates of natural products, such as ANO1 inhibitors schisandrathera D and luteolin.

In conclusion, schisandrathera D inhibited ANO1 channel function and decreased ANO1 protein levels. Schisandrathera D decreased the viability of prostate and oral cancer cells and exerted its anticancer effects by activating apoptosis via the reduction of ANO1 protein levels. However, the anticancer effect was insignificant in cells not expressing ANO1. Similar results were obtained for Ani9, an inhibitor of ANO1 function. Further structure-activity relationship studies should be carried out to develop safer and superior ANO1 inhibitors based on the structure of schisandrathera D. Nevertheless, the results of this study suggest schisandrathera D as a promising lead compound for the development of oral and prostate cancer therapeutic targets which act by targeting ANO1.

## Data Availability

The original contributions presented in the study are included in the article/Supplementary Material, further inquiries can be directed to the corresponding authors.

## Abbreviations

ANO1, anoctamin 1; BSA, bovine serum albumin; CFTR, cystic fibrosis transmembrane conductance regulator; FRT, Fisher rat thyroid; KO, knock-out; MM-GBSA, molecular mechanics–generalized 396 Born surface area; TMEM16A, transmembrane protein 16A; YFP, yellow fluorescent protein.
