# Supplementary material for: ANO1-downregulation induced by schisandrathera D: a novel therapeutic target for the treatment of prostate and oral cancers
Source: Front Pharmacol. 2023 May 18;14:1163970. doi: 10.3389/fphar.2023.1163970 (PMC10232832; doi:10.3389/fphar.2023.1163970)
Supplement: Supplementary file 1 [file DataSheet1.pdf]

# *Supplementary Material*

# ANO1-Downregulation Induced by Schisandrathera D: A Novel Therapeutic Target for the Treatment of Prostate and Oral Cancers

**SeonJu Park[1], Raju Das[2], Nguyen Xuan Nhiem[3,4], Sung Baek Jeong[5], Minuk Kim[6], Dongguk Kim[6], Hye In Oh[7], Su-Hyeon Cho[1], Oh-Bin Kwon[5], Jae-Hyeog Choi[5], Chul Soon Park[8], Song-Rae Kim[1], Uk Yeol Moon[5], Boksik Cha[5], Dong Kyu Choi[5], Sungwoo Lee[5], Wan Namkung[9], Joohan Woo[2,10*] and Yohan Seo[5*]**

*** Correspondence:** Yohan Seo : yohanseo@kmedihub.re.kr; Joohan Woo : lsjoohan@gmail.com

**Supplementary Table S1:** Interaction analysis of ANO1 with Schisandrathera D and Ani9

<table>
<tr><th>Compound name (Docked with ANO1)</th><th>Hydrogen bonds (Distance in Å)</th><th>Hydrophobic interactions (Distance in Å)</th><th>π stacking</th><th>Halogen bond</th></tr>
<tr><td rowspan="5">Schisandrathera D-ANO1 (PDB ID: 5OYB)</td><td>His$^{650}$ (1.84)</td><td>Ala$^{697}$ (3.85)</td><td rowspan="5"></td><td rowspan="15">Asn$^{647}$ (3.70)</td></tr>
<tr><td rowspan="2">His$^{661}$ (1.79)</td><td>Pro$^{701}$ (3.32)</td></tr>
<tr><td>Pro$^{701}$ (3.53)</td></tr>
<tr><td rowspan="2">His$^{695}$ (1.82)</td><td>Lys$^{741}$ (3.59)</td></tr>
<tr><td>Leu$^{746}$ (3.90)</td></tr>
<tr><td rowspan="3">Schisandrathera D-ANO1 (PDB ID: 6BGJ)</td><td>Asn$^{647}$ (3.02)</td><td rowspan="3">Glu$^{701}$ (3.58)</td><td rowspan="3"></td></tr>
<tr><td>Glu$^{701}$ (1.99)</td></tr>
<tr><td>Lys$^{737}$ (2.13)</td></tr>
<tr><td rowspan="2">Ani9-ANO1 (PDB ID: 5OYB)</td><td rowspan="2"></td><td rowspan="2">Leu$^{699}$ (3.71)</td><td>Lys$^{327}$ (4.41)</td></tr>
<tr><td>Lys$^{574}$ (3.97)</td></tr>
<tr><td rowspan="5">Ani9-ANO1 (PDB ID: 6BGJ)</td><td rowspan="5"></td><td>Leu$^{543}$ (3.97)</td><td rowspan="5"></td></tr>
<tr><td>Leu$^{639}$ (3.73)</td></tr>
<tr><td>Leu$^{643}$ (3.50)</td></tr>
<tr><td>Glu$^{701}$ (3.80)</td></tr>
<tr><td>Ile$^{704}$ (3.52)</td></tr>
</table>

**Supplementary Table S2:** Molecular docking and molecular mechanics – generalized Born surface area scores

| ANO1 PDB ID | Docking score | | MM-GBSA score | |
|---|---|---|---|---|
| | 5OYB | 6BGJ | 5OYB | 6BGJ |
| Schisandrathera D | –5.042 | –5.589 | –29.10 | –41.78 |
| Ani9 | –4.095 | –4.612 | –33.08 | –29.10 |

*Energy unit is kcal·mol$^{-1}$.
Legend: PDB, Protein Data Bank; MM-GBSA, molecular mechanics – generalized Born surface area
